# Gut microbiota plasticity in insular lizards under reversed island syndrome

**DOI:** 10.1038/s41598-022-16955-0

**Published:** 2022-07-25

**Authors:** Maria Buglione, Ezio Ricca, Simona Petrelli, Loredana Baccigalupi, Claudia Troiano, Anella Saggese, Eleonora Rivieccio, Domenico Fulgione

**Affiliations:** 1 Department of Biology, University of Naples Federico II, Via Cinthia 26, 80126 Naples, Italy; 2 Task Force of the Federico II University for Microbiome Studies, Naples, Italy; 3 Department of Molecular Medicine and Medical Biotechnology, University of Naples Federico II, Naples, Italy; 4 Department of Humanities, University of Naples Federico II, Naples, Italy

**Keywords:** Evolution, Microbiome, Herpetology

## Abstract

Animals living on small islands are more drastically exposed to environmental changes, such as food or water starvation, and rapid temperature shifts. Facing such conditions, and probably thank to adaptive plasticity mechanisms, some animals display a Reversed Island Syndrome (RIS), a suite of traits, including skin pigmentation, voracity, sexual dimorphism, showed differently from mainland relatives. Here, we analyse a so far poorly explored aspect of RIS: the effect of this on the microbiota composition of host Italian wall lizard (*Podarcis siculus*), strongly influenced by the animal’s lifestyle, and conditioning the same. We compare mainland and island populations, assessing the difference between their microbial communities and their response under unexpected food, experimentally provided. Our observations showed a significant difference in microbiota communities between island and mainland groups, depended mainly from changes in relative abundance of the shared genera (difference due to decrease/increase). Exposure to experimental diet regimes resulted into significative reshaping of bacterial composition of microbiota and a greater variation in body mass only in the island population. Our results could be an evidence that gut microbial community contributes to adaptive plasticity mechanisms of island lizards under RIS to efficiently respond to unexpected changes.

## Introduction

The association between animals and microorganisms living in their gastrointestinal tracts depends on several different factors, such as host genotype, gut morphology, physiological status, immune system, social interaction^1–7^ and environmental inputs^1–4^. However, at the same time the microbiota could impact the ecology of their hosts, influencing their behaviour^5,6^, pathogen resistance^7–9^, reproductive isolation and metabolism^10^. Understanding this paradigm, which defines how the microbiota is both a cause and a consequence of the etho-ecology of a species, can help to decode the life history of vertebrates in different niches and to understand the resulting biodiversity^11^.

Furthermore, adaptive variables and neutral processes, such as drift and dispersal, could also induce large part of animal intraspecific microbial variation^12–14^. This is a particularly interesting topic for investigations on vertebrate populations living on islands. In fact, separation of an island population from the mainland origin can act over time and shape the microbiota compositional structure, maintaining/increasing microbial similarity among populations through microbiota inheritance^15^, or driving microbiota divergence among populations through selective and stochastic changes in taxa relative abundances^16^ and/or acquisition of novel taxa from the local conditions^17^.

An interesting island model system is represented by populations of lizards living on small islands close to the mainland, undergoing the Reverse Island Syndrome (RIS)^18,19^ for which rapid phenotypic changes are often visible in a short time^20^, in our interpretation, due to genome plasticity (sensu^21^) based on differential expression of some genes^22,23^.

The RIS infers that the lizard populations from islets, generally close to mainland, living under unpredictable environmental conditions, such as high predation pressure, mortality risk and chances of catastrophe, often exhibit a suite of traits different from populations living on the mainland. Among these traits, we found more aggressive behaviour, higher food intake rate, increased energy allocation for reproduction, an early sexual maturity time, and melanic colouration. All of this in order to increase the chance to invest in the next generation^18,19^. However, a so far poorly explored aspect of the syndrome is the relationship with the microbiota of host animals.

The vertebrate gut microbiota is known to be highly plastic and able of changing in response to environmental variation, in order to facilitate animal adaptation to the new conditions^24^. For example, gut microbial community structure in lizards depends heavily from the immune system^25^, which is one of the most relevant traits affecting by the RIS^18,19,26^. Furthermore, the gut microbiota is an important driver of parasite resistance^27,28^, and influences behavioural aspects, such as hyperactivity and aggressiveness^29^ as well as the ability to obtain and store more energy from the diet, contributing to body-weight gain^30^. Moreover, some studied reported that island populations to over-come dietary limitations, could expand their feeding preferences and/or maximize energy acquisition^31,32^.

**Figure 1:**
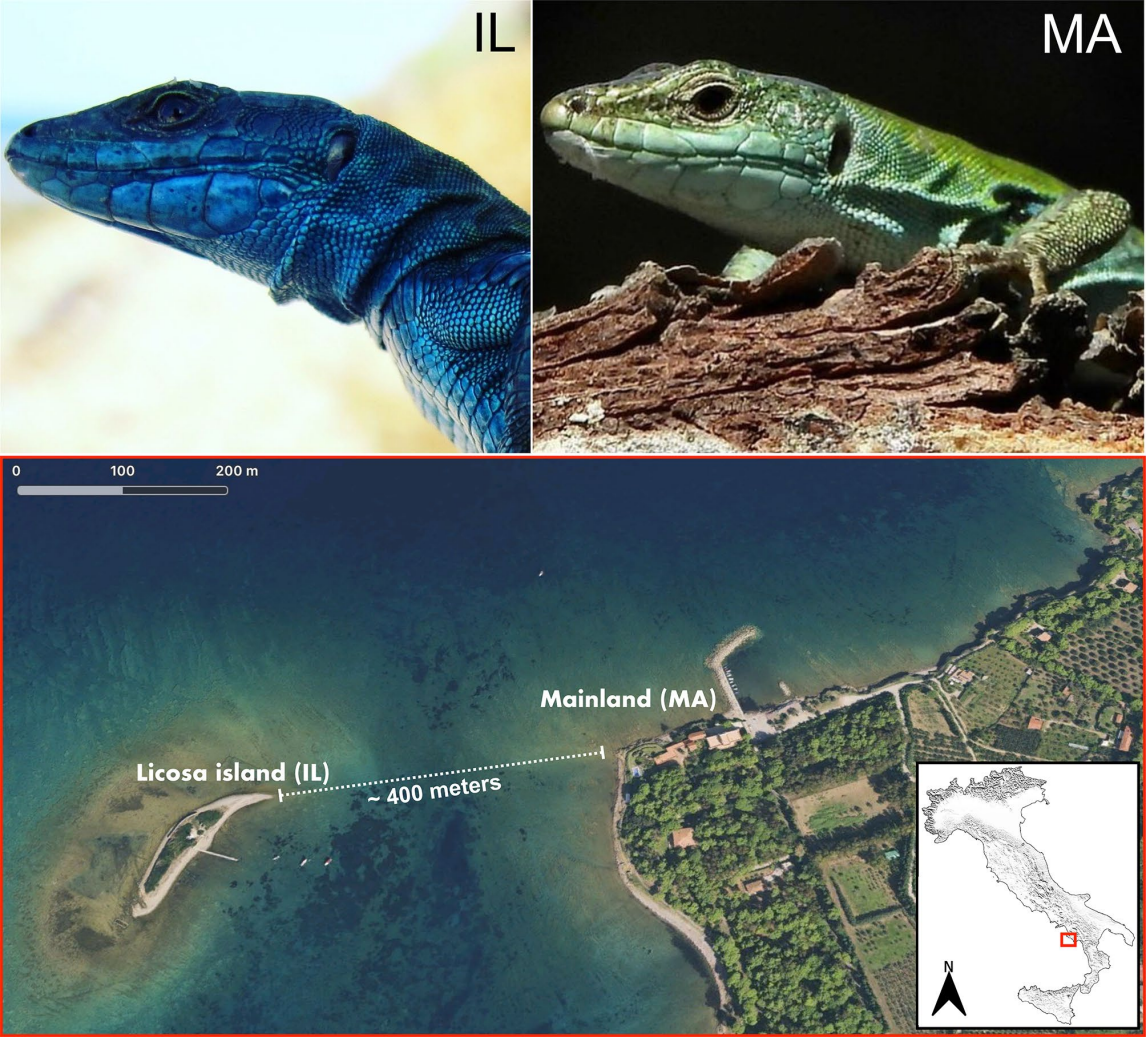
Study area and lizard phenotypes. Lizards from island (IL) and mainland (MA). Licosa islet (40°15′N, 14°54′E, Salerno, South Italy), about 400 m far from the close mainland (Punta Licosa, 40°50′N, 14°15′E, Salerno, South Italy). Satellite image was obtained from Maps version 2.1. 2012–2018 Apple Inc. The photos of animals were taken by Domenico Fulgione.

Considering the link between the microbiota and different aspects involved in the RIS, we asked if this syndrome could have an impact on the composition and plasticity (sensu^21^) of the gut microbial communities of host island lizards. To explore this question, we performed a comparative analysis of the gut microbial communities considering a blue melanic population of the Italian wall lizard (*Podarcis siculus klemmeri*)^33^, endemic to Licosa islet (Salerno, South Italy), affected by the RIS, and their continental relatives (*Podarcis siculus siculus*)^34^ on the overlooking mainland, with common green back and white belly (Fig. 1). The island population certainly originated from the facing mainland no more than 4000 years ago^35–37^, the presumably dated origin of the island^38^. The current taxonomic classification reported the island lizard as a subspecies (*Podarcis siculus klemmeri*^33^), based mainly on its blue colouration. The short distance between the island and the mainland allows for the existence of a moderate gene flow between the two populations, excluding important phenomena of genetic drift^38^. Therefore, we believe that this character is caused by the RIS as reported in some of our papers^18,19,22,23^.

Here, to speculate about plasticity, the causal role and/or consequences on a microbiota affected by rapid and unexpected changes, we tested what happens to the microbiota composition of lizards under an experimental diet variation. Therefore, we set up cage trials of food composition alteration to test if and how the gut microbiota of the two populations was able to respond.

## Results

Starting from 43 sampled lizards, 35 were useful to analyse the gut bacterial composition, selecting those showing the best quality parameters of extracted DNA (> [50 ng/μL]; λ_260/280_ > 1.70; λ_260/230_ > 1.70): fasting island lizard, IL = 8; fed island lizard, IL_F = 10; fasting mainland lizard, MA = 9 and fed mainland lizard, MA_F = 8).

During experimental period, fed island lizards ate in average 19.54 ± 6.5 worms while fed mainland lizards consumed 16.00 ± 13.51 worms in average.

### Sequencing reads report

A total of 5,070,956 short raw reads (35–290 bp) were obtained from Illumina MiSeq sequencing with an average of 144,884 ± 18,391 reads x sample, and of 144,635 ± 3,128 reads × group (IL: 141,951 ± 27,193; IL_F: 149,052 ± 12,073; MA: 144,523 ± 18,831; MA_F: 14,3015 ± 16,491) (Supplementary Table S1 online). The raw reads were separately processed and 2,016,960 filtered sequences (mean × sample ± SD: 57,627.43 ± 14,765.14) resulted after the bioinformatics analysis of the data, reaching 1,965,572 sequences (mean × sample ± SD: 56,159.20 ± 14,423.55) considering only the non-chimeric ones (Supplementary Table S1 online). The analysis of blank-negative water samples and mock communities controls did not reveal any inconsistencies in the expected profiles.

The Observed OTUs, Chao1, and Shannon rarefaction curves reached the plateau for all samples, showing that the estimates of species richness were stable and unbiased and the sequencing depth was sufficient for capturing a majority of microbial diversity and differences in microbial communities in the samples (Fig. S1a–c, Supplementary information online).

### Microbiota composition of the island and mainland populations

Comparing island (IL) and mainland (MA) microbiota of fasting lizards, taxonomic assignment revealed 9 and 10 bacterial phyla for island IL and mainland MA lizard microbiota, respectively. In particular, Firmicutes were on average the most represented phylum (IL: 49.00% ± 0.16; MA: 38.20% ± 0.12) both for island and mainland lizards, followed by Bacteroidetes (IL: 23.15% ± 0.12; MA: 30.92%, ± 0.11) and Proteobacteria (IL: 15.82% ± 0.14; MA: 23.51% ± 0.14) meanwhile Patescibacteria was missed in IL (Supplementary Table S2 online).

A greater variation is pointed out in terms of number of bacterial families comparing microbiota of the two populations (IL: 42 families *vs* MA: 52 families). In particular, Bacteroidaceae (IL = 10.74% ± 0.07; MA = 16.28% ± 0.07)*,* Enterobacteriaceae (IL = 14.08% ± 0.13; MA = 15.01% ± 0.15)*,* Lachnospiraceae (IL = 29.08% ± 0.13; MA = 15.00% ± 0.10) and Ruminococcaceae (IL = 9.80 ± 0.03; MA = 11.34% ± 0.07) were the most represented families, followed by Tannerellaceae in mainland lizards (IL = 5.0 ± 0.02; MA = 7.22% ± 0.04), and Akkermansiaceae in island lizards (IL = 7.94% ± 0.1; MA = 2.87% ± 0.02) (Supplementary Table S3 online).

**Figure 2:**
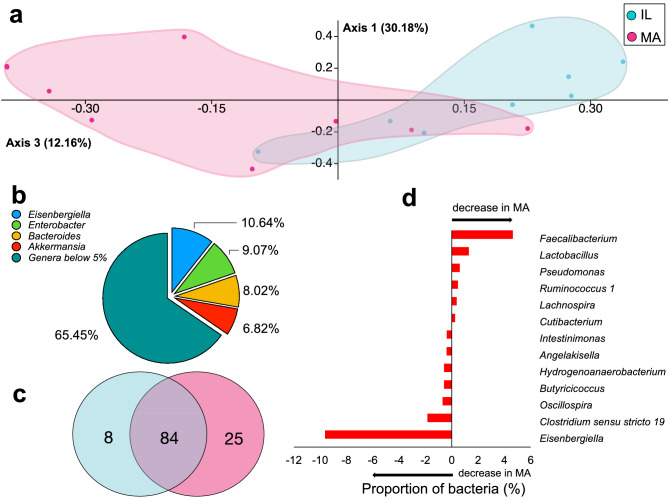
Comparison between island and mainland lizards using the gut microbiota community. (**a**) Principle Coordinate Analysis (PCoA) using Bray–Curtis dissimilarity to test β-diversity between the gut microbiota of IL (island) and MA (mainland) lizards. (**b**) Contribution of bacterial genera to differences among the gut microbiota of IL (island) and MA (mainland) lizards calculated using SIMPER (Similarity Percentages Species Contributions); see Supplementary Table S4 online for details. (**c**) Symmetric Venn diagram of shared and exclusive bacterial genera in the gut microbiota of IL (island) and MA (mainland) lizard groups, produced by Venn Diagram Tool freely available on the web (https://bioinformatics.psb.ugent.be/webtools/Venn/); see Supplementary Table S5 online for details. (**d**) Variation of shared bacterial genera showed as the amount of bacteria (%) in the gut microbiota of mainland (MA) compared to island (IL) lizards.

The two fasting populations differed significantly (one-way permutational multivariate analysis of variance, PERMANOVA test, *p*: 0.003) considering the bacterial communities at the genus taxonomic level. Indeed, Principal Coordinates Analysis (PCoA) accounted for 42.34% of variance, taking into account the first and the third axes (Fig. 2a). The ordination by this analysis produced two separate clusters, whose difference depended most of all from the contribution of *Eisenbergiella* (contribution of 10.64%), *Enterobacter* (9.07%), *Bacteroides* (8.02%) and *Akkermansia* (6.81%), with 60.07 of overall average dissimilarity (Fig. 2b and Supplementary Table S4 online). Furthermore, they shared 84 bacterial genera, with 8 and 25 exclusive for the gut microbiota of island lizards and mainland lizards, respectively (Fig. 2c and Supplementary Table S5 online).

The gut microbiota of IL lizards showed 92 bacterial genera, of which *Eisenbergiella* (13.86% ± 0.08) and *Bacteroides* (11.05% ± 0.07) were on average the most abundant ones, meanwhile for the MA gut microbiota, on 109 genera identified, *Bacteroides* (16.22% ± 0.08), and *Enterobacter* (10.04% ± 0.13) resulted to be on average the most abundant ones. All of the other genera showed an average occurrence lower than 10% (Supplementary Table S6 online).

Comparing MA *vs* IL microbiota of fasting lizards by a post-hoc analysis, a total of 21 genera were found to be differentially represented, with 6 genera only present in the gut microbiota of mainland animals and 2 genera exclusively present in the gut microbiota of island lizards. These exclusive genera included Gram-negatives (*Prevotella*, *Zooglea*, *Rivicola*, *Sulfuricum*, *Dysgonomonadaceae*) and Gram-positive (*Streptococcus*, *Eubacterium eligens*, *Pelosinus*) bacteria (Table 1).

**Table 1:** Bacterial genera exclusive in mainland (MA) and island (IL) microbiota.

Genus	Relative abundance (mean % ± SD)	
	MA	IL
*Eubacterium eligens group* (*Lachnospiraceae family*)	0.62 ± 0.61	–
*Prevotella 9*	1.61 ± 1.63	–
*Streptococcus*	0.28 ± 0.31	–
*Zoogloea*	0.68 ± 0.86	–
*Sulfuricurvum*	0.27 ± 0.31	–
*Rivicola*	0.14 ± 0.22	–
*U.m. Disgonomonadaceae family*	–	0.34 ± 0.41
*Pelosinus*	–	0.52 ± 0.53

Relative abundance (mean %) and standard deviation (SD) of bacterial genera present exclusively in mainland (MA) and island (IL) microbiota.

The 13 shared genera differentially represented in the two groups were *Faecalibacterium*, *Lactobacillus*, *Pseudomonas*, *Ruminococcus* *1*, *Lachnospira* and *Cutibacterium*, more abundant in the gut microbiota of MA lizards, and *Intestinimonas*, *Angelakisella*, *Hydrogenoanaerobacterium*, *Butyricicoccus*, *Oscillospira*, *Clostridium *sensu stricto* 19* and *Eisenbergiella*, more represented in IL animals (Fig. 2d). The Pearson’s chi-square test, performed considering all 21 genera and permutation with 9999 replicates, indicated that observed differences between IL and MA groups were independent from random variable (Chi-square value: 11.415, degrees of freedom: 4, *p* value: 0.002). Furthermore, this was confirmed by Fisher´s exact test (*p* value: 0.003).

### Variation of gut microbiota communities’ composition under unexpected food change

To characterize the plasticity of island and mainland lizards, we imposed an unusual diet, and then compared the gut microbiota composition of IL *vs* IL_F (moth larvae-fed island lizards) and MA *vs* MA_F (larvae-moth fed mainland lizards) groups.

**Figure 3:**
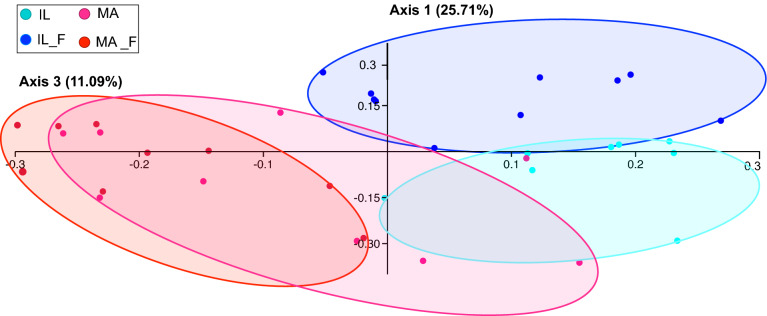
Comparison between the microbiota communities of lizards before and after food supplied. Principal Coordinate Analysis (PCoA) using Bray–Curtis dissimilarity to test β-diversity among the gut microbiota of IL (island lizard), IL_F (fed island lizard), MA (mainland lizard) and MA_F (fed mainland lizard) samples.

After the same experimental treatment, both microbiota of fed island lizards (IL_F) and fed mainland lizards (MA_F) rearranged in a different way compared with the original corresponding microbiota community (fasting IL and MA, respectively), however statically significative difference was revealed only for insular microbiota (Fig. 3). In particular, PCoA generated using the bacterial genera of the four groups accounted for 36.80% of variance (Fig. 3), with microbiota of island lizards that split significantly from microbiota of fed animals (one-way PERMANOVA, *p* < 0.001; see Supplementary Table S7 online for details), whereas MA_F was almost completely overlapping MA in the plot (one-way PERMANOVA, *p*: 0.27).

**Figure 4:**
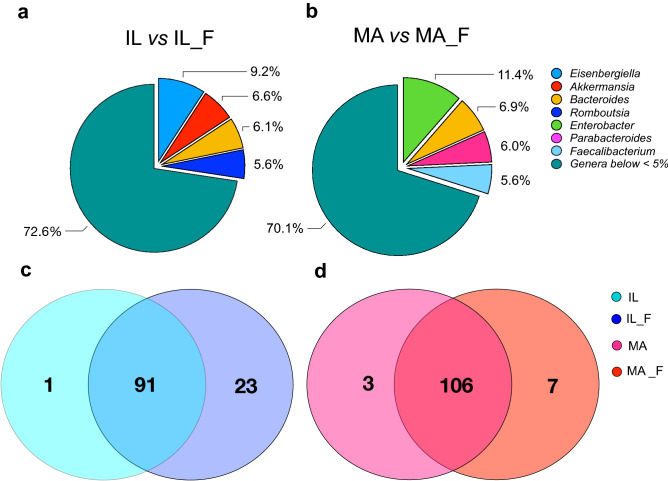
Relative contribution and exclusiveness of bacterial genera in the gut microbiota communities. Contribution of bacterial genera to differences among the gut microbiota of (**a**) IL (island lizard) *vs* IL_F (fed island lizard), and (**b**) MA (mainland lizard) *vs* MA_F (fed mainland lizard), calculated using SIMPER (Similarity Percentages Species Contributions). See Supplementary Table S8 and Supplementary Table S9 online for details. Symmetric Venn diagram of shared and unique bacterial genera in the gut microbiota of (**c**) IL (island lizard) *vs* IL_F (fed island lizard) and (**d**) MA (mainland lizard) *vs* MA_F (fed mainland lizard) groups, produced by Venn Diagram Tool freely available on the web (https://bioinformatics.psb.ugent.be/webtools/Venn/). See Supplementary Table S10 and Supplementary Table S11 online for details.

The Similarity Percentages Species Contributions (SIMPER) analysis considering IL and IL_F populations (Fig. 4a) showed an overall average dissimilarity of 59.93, with *Eisenbergiella* (contribution of 9.36%), followed by *Akkermansia* (6.58%), *Bacteroides* (6.12%) and *Romboutsia* (5.04%), that were the most involved genera in the differentiation between microbiota of the island groups (Supplementary Table S8 online). Meanwhile, *Enterobacter* (11.4%) was the genus mainly involved in determining the dissimilarity between MA and MA_F groups (Fig. 4b and Supplementary Table S9 online).

At the phylum level, intra-groups variations (IL *vs* IL_F and MA *vs* MA_F) concerned the average relative read abundance rather than the type and number of phyla, except for the microbiota of IL_F lizards, showing an extra phylum (Patescibacteria) compared to IL lizards (N_IL_ = 9, N_IL_F_ = 10, N_MA_ = 10, N_MA_F_ = 10). The dominance of Firmicutes (IL_F: 41.43% ± 0.16; MA_F: 35.62% ± 0.1), Bacteroidetes (IL_F: 28.39% ± 0.10; MA_F: 32.17% ± 0.09) and Proteobacteria (IL_F: 19.77% ± 0.07; MA_F: 25.36% ± 0.10) was confirmed in both cases (Supplementary Table S2 online).

A similar pattern could be highlighted at the family level, considering both the average relative read abundance, and the type and number of taxa (Supplementary Table S3 online). Indeed, in this case too, after supplying food, only IL_F lizards gained 10 more families than fasting IL lizards (N_IL_ = 42, N_IL_F_ = 52, N_MA_ = 52, N_MA_F_ = 52). Lachnospiraceae (IL_F = 14.26% ± 0.007; MA_F = 10.81% ± 0.03), Enterobacteriaceae (IL_F = 11.63% ± 0.07; MA_F = 12.55% ± 0.08)*,* and Bacteroidaceae (IL_F = 9.8% ± 0.04; MA_F = 16.02% ± 0.05) turn out to be the most represented families once more (Supplementary Table S3 online).

Even at the genus level, the variation showed in the island system concerned both the type, the number and the relative abundance of bacterial genera (Supplementary Table S6 online). Indeed, of 115 total bacterial genera, microbiota of IL and IL_F lizards shared 91 genera, and only one (*Candidatus Stoquefichus*) was exclusive of fasting lizards, while 23 were unique of fed island samples (Fig. 4c and Supplementary Table S10 online). This trend was also found in the mainland lizard groups even if less evidently. Indeed, of 116 genera, 106 were shared between the microbiota of MA and MA_F lizards, 3 genera were exclusive of microbiota of MA lizards and 7 were exclusive of microbiota of MA_F group (Fig. 4d and Supplementary Table S11 online).

**Figure 5:**
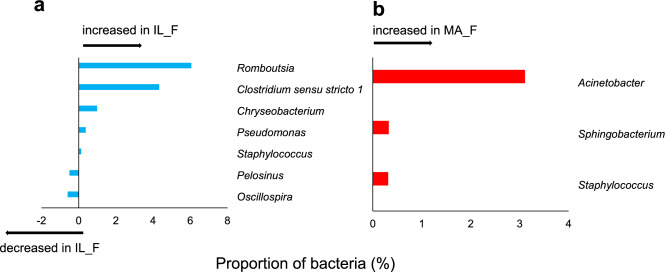
Differentially represented genera among the gut microbiota. Differentially represented genera (amount of bacteria %) among the gut microbiota of (**a**) IL (island lizard) *vs* IL_F (fed island lizard) and (**b**) MA (mainland lizard) *vs* MA_F (fed mainland lizard).

*Bacteroides* was on average the most abundant genus both for microbiota of IL_F (9.73% ± 0.04) and MA_F (15.96% ± 0.04) lizards (Supplementary Table S6 online). A quantitative analysis showed a significant change in 8 genera differentially represented in IL *vs* IL_F (Fig. 5a) and only 3 in MA *vs* MA_F (Fig. 5b) lizards.

Comparing shared genera in the microbiota of IL *vs* IL_F lizards, 5 genera (i.e. *Romboutsia, Clostridium *sensu stricto* 1, Chryseobacterium, Pseudomonas* and *Staphylococcus*) increased in IL_F animals and 2 genera (*Pelosinus* and *Oscillospira*) increased in IL ones (Fig. 5a).

The 3 genera differentially represented in the microbiota of MA *vs* MA_F groups (*Acinetobacter, Sphingobacterium* and *Staphylococcus*) were all more abundant in MA_F than in MA (Fig. 5b). Interestingly, members of the *Sphingobacterium* and *Staphylococcus* increased both in IL_F and MA_F groups suggesting a direct link between their abundance and the specific diet.

**Figure 6:**
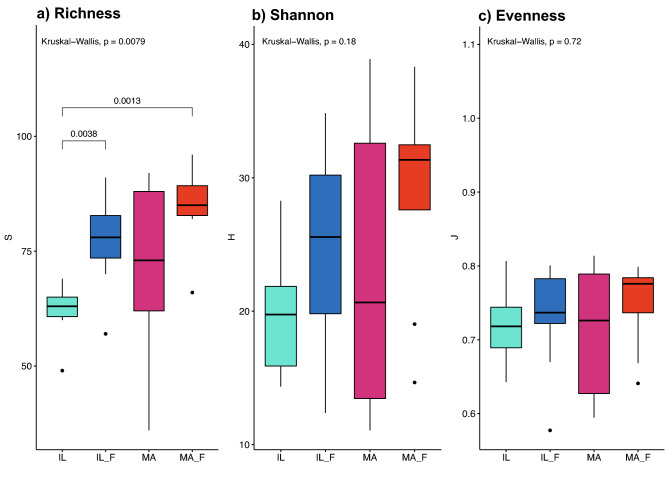
α-Diversity descriptors in the gut microbiota communities. (**a**) Richness (S) (**b**) Shannon (H) and (**c**) Evenness (J) for the gut microbiota of IL (island lizard), IL_F (fed island lizard), MA (mainland lizard) and MA_F (fed mainland lizard). Square brackets indicate statistically significant differences.

Analysis of the α-diversity descriptors, calculated at genus taxonomic level, indicated that the unusual diet caused an increased microbial diversity both for the microbiota of IL_F and MA_F lizards (Fig. 6), although statistically significant differences were observed only for Richness (Kruskal–Wallis, *p*: 0.007) between IL and IL_F groups (H: 8.597, *p*: 0.003) and IL and MA_F (H: 10.599, *p*: 0.001).

### Weight adjustment

**Figure 7:**
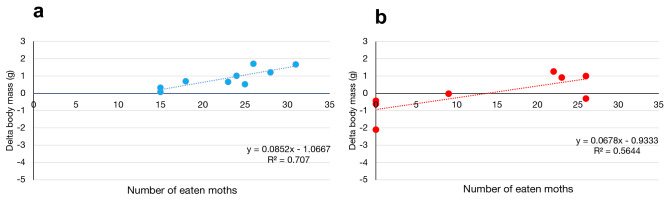
Weight increase after food suppling. Correlation between the number of ingested moths and weight change (g) in (**a**) island and (**b**) mainland lizard groups.

During experimental period, fed island lizards and mainland lizards ate a comparable number of moth larvae (mean ± SD: 19.54 ± 6.5 *vs* 16.00 ± 13.51; not significant difference by ANOVA test, *p*: 0.47). It is interesting to note that this food surplus provided to both populations generates a greater variation in body mass only for island lizards, probably as a result of a reorganization of their microbiota based on this unexpected food source (Fig. 7). Only a single fed island sample (IL_35_F) showed a reduction in weight after the experimental treatment (delta body mass: − 4 g). Thus, we discarded it from this analysis by attributing this distortion to an error in weight measurement. Nevertheless this, the correlation between weight variation and ingested moths was always higher for fed island lizards (R^2^ 0.707; t-score: 4.11; *p*: 0.004) than for mainland lizards (R^2^ = 0.5644; t-score: 2.788; *p*: 0.03) (Fig. 7).

## Discussion

Our experimental model turns out to be optimal for revealing how RIS affects the possible changes of the gut microbiota community of lizards, as testified to the significative differences between microbiota composition of IL and MA.

Variation in microbiota of lizards under experimental condition may depend on diet (i.e. omnivorous *vs* herbivorous) and host species^2^. Our populations belong to the same omnivorous species and were collected during the same season, thus, we could assume that factors as (i) the introduction of “food” as a sudden and unexpected change, (ii) the simplification of the diet in terms of number of ingested items and, (iii) the fasting, affect the variation in microbiota of our experimental groups. It is interesting, and worthy of further study, that these constraints introduce an increase in microbiota diversity.

Gut microbial composition results from a mix of commensal “core” genera (sensu^39,40^) and a flexible pool of beneficial microbes which may confer a selective advantage during periods of stress or rapid adaptation^41^. The analysis of the latter component can be an indicator of the adaptive potential of a species or a population. It allows to speculate on plasticity and ecological segregation, particularly for species that are unable to make large displacements and subjected to the ecological conditions where they are confined.

Our findings showed that the microbiota diversity depends mainly on different relative abundances of the shared genera (difference due to decrease/increase) rather than changes in bacterial genera composition (difference due to substitution). For example, the gut microbiota of mainland and island lizards, although characterized by a different number of bacterial genera (92 *vs* 109, respectively), shared a core of 84 genera, showing similar values of alfa-diversity index. All this was affected probably by the dominance of few bacteria (*Bacteroides* and *Enterobacter* for MA lizards and *Eisenbergiella* and *Bacteroides* for IL lizards) characterized by the highest average relative abundance, followed by all the other genera showing an average relative abundance less than 10%.

The gut microbiomes of squamate reptiles have been largely overlooked in terms of ecology^42^ when compared to the other vertebrates^43^. Indeed, less than 10% of studies investigating the gut microbiota communities of vertebrates were conducted on non-mammalian hosts^42^ and some of these indicate that squamate reptile microbiota would be more similar to those of fishes and birds than those of mammals^43^.

In our study, the more abundant genera in IL lizards belong to four Families: Oscillospiraceae  (*Hydrogenoanaerobacterium*, *Oscillospira*), Ruminococcaceae  (*Intestinimonas*, *Angelakisella*), Clostridiaceae  (*Butyricicoccus*, *Clostridium sensu stricto 19*) and Lachnospiraceae  (*Eisenbergiella*). In particular, *Oscillospira* is commonly found in the intestinal microbiota of vertebrates with a potential role in the digestion of cellulose and in the fermentation of fibres in herbivorous, including reptiles^42,44–46^. All members of the *Oscillospira* genus have been shown to use animal-derived glycans (i.e. glucuronate) to produce butyrate^47^. The abundance of *Oscillospira* increased in response to prolonged fasting in lizards and other vertebrates as well^48^, probably promoting the degradation of some glycans (i.e. fucose, sialic acids, and glucuronic acid) of the host^48^. Accordingly, in our study, we observed an increase of *Oscillospira* in fasting island lizards compared to fed island lizards (delta-variation: 0.006). This probably represents a response to the unpredictable conditions on the island that lead these lizards to cope with periods of prolonged fasting.

MA populations showed lower level of *Oscillospira* than IL, and this decreased after food providing but with slight variation if compared to IL microbiota. Arguably, the differential level of *Oscillospira* and variations in a shorter time interval than mainland conspecifics are in agreement with RIS^23^.

Members of the *Intestinimonas* and *Angelakisella* are also not known in detail, however they are considered specialized in the digestion of cellulose with an essential role in the fermentation of fibres in herbivores, including reptiles^2,44^. This could be related to a greater herbivorous behaviour in island lizards to survive during periods of low animal prey availability.

The genus *Clostridium sensu stricto*, anaerobic Gram-positive spore formers commonly found in human and animal guts^49^, and *Eisenbergiella*, isolated from human blood and faecal samples^50^, were found involved in the maintenance of gut homeostasis and in modulating the functional activities of the cells of the immunological system^49^. In particular, the latter was strongly correlated with increased levels of TNFα and IFNα in chickens’ intestinal epithelial cells^51,52^. All of this is in line with the RIS that predicted a more active immune system in island lizard than in mainland ones^19^.

Bao and co-workers reported an increase in *Eisenbergiella* after infection by *Echinococcus granulosus* in mice^53^. Furthermore, Dipineto and collaborators, analysing parasites in lizard populations on mainland and island from the same as our study area, showed the presence of Coccidae only in faeces of island lizards^54^. Interestingly, our results indicated that the relative abundance of *Eisenbergiella* is significantly greater in the microbiota of IL than MA.

Among the bacterial genera more significant discriminative of MA lizards microbiota we found *Enterobacter*, ubiquitous^55^ and also part of the commensal microflora of animals^56^ and of the human gut^57,58^. The microbiota of fasting MA lizards showed relative abundance of *Enterobacter* higher than fasting IL lizards. A possible explanation for this could be that mainland lizards have more opportunities of contacting with humans or animal (wild and domestic) waste rather than the island lizards. Indeed, the latter lives on Licosa islet, only occasionally frequented by birds or humans to swim or boat during the summer. This hypothesis is according to the isolation condition that affect lizards under RIS and opens up interesting hypotheses on the synanthropic^59^ commensalism of this animal.

In our study, we did not perform a direct characterisation of microbial community of moth. However, to select which bacteria could be introduced by the food supplied, we have extracted the items shared uniquely by the fed lizards and absent in island and mainland fasting ones, that were *Candidatus* and *Rickettsiella*.

The former characterises the microbial community of vertebrates (i.e.^60^), and therefore we excluded that its presence in gut microbiota of lizards was due to a conditioning by the microbiota of the moth.

*Rickettsiella* was found in a great variety of arthropod species^61–63^, including also some parasites adherent to tissues of *Podarcis* sp.^64^. Therefore we cannot define with certainty whether this bacterial genus is part of the microbiota of lizards, whether it derives from the infection of these by parasites or from the diet provided in captivity. Nevertheless, *Rickettsiella* genus was extremely underrepresented in the microbiota community of fed mainland (mean ± SD: 0.0000053 ± 0.000102) and fed island lizards (mean ± SD: 0.01 ± 0.030). Further investigations into the ecology of allochthonous microbes would provide more insight into the assembly of the gut microbiota of lizards.

The supply of unexpected food changed, qualitatively and quantitatively, the microbiota composition both of mainland and island populations; however, 91 and 106 bacterial genera were still shared between fasting and fed lizards, in insular and mainland intra-comparation, respectively (Fig. 4c,d). This result further suggests that the lizards maintained their core gut microbial communities during the 5 days of experiment, as demonstrated also by Kohl et al.^2^ that showed how their captive lizards, treated for 8 weeks, retained ~ 65% of their wild bacterial microbiota. Probably, the consistency of this core will reduce over time, and the faster variation that we detected could be explained by the syndrome that island lizards undergo.

Interestingly, comparing the microbiota composition of fasted and fed lizards, we revealed statistically significant differences only for island populations. This variation could be more evident for island system because of the lower richness in bacterial genera of the microbiota compared to that of mainland lizards (15% less). However, it should also be considered that these two groups shared 77% of the microbial bacterial groups. These results could be an evidence of the ability of the island lizards (under RIS) to respond more significantly than mainland populations to drastic and unexpected changes, by adopting adaptive plasticity mechanisms^11,21^, adding another important element to the theoretical basis of the syndrome^18,19,22,23^.

Our contribution opens the doors to future investigations aiming at shedding light on a mechanism as interesting as the plasticity of populations in highly unpredictable environmental conditions, such as those on small islets, revealing how gut microbial communities may be impacting the ecology and evolution of island lizard hosts.

Probably, the attitude of island lizards to have a high feed intake (according to the RIS) and to use a variety of food items, such as the regurgitation of gulls (personal observation), could explain plasticity observed in the microbiota of island individuals.

Furthermore, the island lizards showed to be bolder than mainland relatives (according to the RIS) and, thanks to this, they may have the opportunity to explore more complex ecological niches. This circumstance could promote a modelling of the microbiota in relation to the resources encountered. These etho-ecological peculiarities of the island lizards could be linked to their microbiota, which would be better adapted to exploit the unexpected resources, as suggested by the weight increase recorded in the experimental group (IL_F).

Moreover, there are probably key genes involved in the plasticity and peculiarity of the microbiota of insular lizards. We previously described the transcriptome of *P. siculus*^22^ considering mainland and island specimens, and this could represent an opportunity to deep the information about differential expressed genes underling host-microbiome interactions.

## Material and methods

### Study area

Our survey was conducted on the islet of Licosa and on the facing mainland, during the summer. The islet of Licosa (40°15′N. 14°54′E. Salerno, South Italy) is 400 m away from the closest mainland (Punta Licosa, 18. 40°50′N. 14°15′E) (Fig. 1) and is dominated by *Pistacia lentiscus* with naked and stony shores. On the island there are small populations of rats (*Rattus* sp*.*), Mediterranean house geckos (*Hemidactylus turcicus*) and very few nesting passerine birds. However, the island predators of lizards are potentially represented by gulls (*Larus michahellis* and *Chroicocephalus ridibundus*), very rarely kestrels (*Falco tinnunculus*), and carrion crows (*Corvus corone*)*.*

The mainland is characterised by luxuriant Mediterranean scrub with olive tree cultivations, rural buildings and stone walls. Mainland lizards are preyed on by mostly birds as tawny owls (*Strix aluco)*, little owls (*Athene noctua*), kestrels, red-backed shrikes (*Lanius collurio)* and carrion crows as well as by terrestrial predators like rats, grass snakes (*Natrix natrix*), green whip snakes (*Hierophis viridiflavus*), and feral cats (*Felis catus*).

The food items of island and mainland populations of lizards were almost similar, consisting mainly of arthropods, such as Diptera, Isopoda and Coleoptera, larvae of Lepidoptera (^31,65–67^ and our field observations) and occasionally of small vertebrates^68,69^.

Moreover, our field observations showed that island lizards supplement their diet with gull regurgitates, chicks of passerines, or they can exploit occasional phenomena such as the migrations of butterfly that stop-over on the island.

### Collection of samples

A total of 43 individuals were caught with nylon loop: 22 on Licosa island (19 males and 3 females) and 21 on the mainland (20 males and 1 female).

The captured lizards were transported to the research station and individually placed in sterile terrariums with autoclaved soil, and kept at 23–27 °C with natural day/night periods. Lizard boxes, accessorized with habitat decorations including wood branches and rocks, were separated with cardboards so the lizards could not see one another, to avoid potential stress due to interaction. Each experimental group of lizards was placed in a separate room.

All lizards used for the analyses were > 1 year old, aged according to the snout-vent length (SVL), which is correlated with age^18,70^. Island lizards had a SVL of 7.5 ± 0.4 cm while SVL for mainland lizards was 7.8 ± 0.3. The lizards were weighed to the nearest 0.01 g on Mettler precision balance (Mettler Toledo) before and after the experimental trials.

The samples were divided into four experimental groups: two lizards groups held without supplied food (fasting island lizards: IL = 9 and fasting mainland lizards: MA = 10) and two lizards groups fed and watered ad libitum for 5 days (fed island lizards: IL_F = 13 and fed mainland lizards: MA_F = 11). Zero time (T_0_) corresponded to the first day of capture of a lizard and, since not all the lizards were caught on the same day, T_0_ does not necessarily synchronized among the specimens.

The water was sterilized under UV lamp for 1 h before the using^71^.

The food was represented by Honeycomb moth (*Galleria mellonella*) caterpillars, not present where lizards lived, bought in a company specializing in laboratory animals. The moth was selected as an exotic diet to evaluate the ability of lizards to adapt to a new dietary regimen.

We used faecal samples as a proxy for gut microbiota considering that they provide a complete view of hindgut bacterial communities in lizards useful for microbial inventories^2,72,73^. In particular, during the experimental period, we monitored the animals at least every 5 h so that we sampled only fresh faecal material (< 5 h). For fed animals, the faecal material deposited in the terrarium soon after catching were discarded. The scats were collected using sterilized equipment and stored immediately in 2 ml microcentrifuge tubes frozen at − 20 °C. Each lizard laid on average 4.13 ± 1.43 excrements in 5 days (in particular, IL = 3.61 ± 1.11; IL_F: 5.13 ± 1.8; MA = 3.61 ± 0.92; MA_F = 3.93 ± 1.14). The faeces of a single individual during the 5 experimental days were pooled every time in the same tube. At the end of experimental period, the pools were transported in containers, at controlled temperature, to the laboratory. Here, they were stored at − 20 °C until DNA extraction and sequencing, performed at most three day after arrival.

Lizards were collected with the authorization of the Ministry of the Environment and Protection of Land and Sea, and experimental procedures, approved also by the institutional review board “Societas Herpetologica Italica”, were performed according to Italian law. This study follows the recommendations reported in the ARRIVE guidelines^74,75^ and all the methods were performed in accordance with the relevant guidelines and regulations. At the end of 5 experimental days for all lizards, they were released at the point of capture.

### DNA extraction

The extraction of DNA from faecal materials was performed in a room dedicated to environmental samples using QIAamp DNA Fast Stool Mini Kit (QIAGEN GmbH Valencia, CA, USA) according to guidelines. Blank extractions were systematically included to check for potential cross-contaminations. DNA quality and quantity were checked using Nanodrop ND-2000 (Nanodrop, Wilmington, DE, USA) and Qubit Fluorometer 3.0 (Thermo Fisher Scientific).

### Microbiota identification by 16S rRNA gene amplification, sequencing, and data analysis

A fragment of about 190 bp of 16S rRNA gene V3 region was amplified using Probio_Uni (5′-CCTACGGGRSGCAGCAG-3′) and Probio_Rev (5′-ATTACCGCGGCTGCT-3′) primers, and then sequenced on Illumina MiSeq platform at GenProbio srl (www.genprobio.com), according to the protocol described in^76^. The sequencing included blank-negative water samples and specific mock communities (ZymoBIOMICS HMW DNA Standard) as additional quality check control.

After demultiplexing, the reads of each sample were trimmed and filtered to remove low quality and chimeras, and processed using a script based on the QIIME software suite^77^. Paired-end reads were assembled to reconstruct the complete Probio_Uni /Probio_Rev amplicons. The sequences between 140 and 400 bp in length and mean sequence quality score > 20 were retained, removing mismatched primers and sequences with homopolymers > 7 bp. 16S rRNA Operational Taxonomic Units (OTUs) were defined at ≥ 99% sequence homology using uclust^78^ and OTUs with less than 10 sequences were filtered. All reads were classified to the lowest possible taxonomic rank using QIIME^77^ and the SILVA database v. 132 clustered at 99% identity as reference dataset^79^.

The results are expressed as percentual frequency for each sample, defined by the ratio between the number of reads in each OTUs and the total number of reads. Representative OTUs of the same genus were added together to have the taxonomic profile at the genus level. Extremely poorly represented taxa (relative abundance < 0.002%) were discarded from the subsequent elaborations, according to^80,81^. Finally, we calculated the number of observed OTUs, Chao1 and Shannon indices.

### Statistical analysis

Community membership and structure were represented by a PCoA using Bray–Curtis dissimilarity^82^. One-way PERMANOVA was performed to test significance of multivariate analyses.

We assessed which taxa are primarily responsible for an observed difference between groups of samples^83^ using SIMPER with Bray–Curtis dissimilarity, in Past v. 3.2. software^84^.

Sample α-diversity was calculated using Richness^85^, Evenness^86^ and Shannon’s^86^ in Past v. 3.2. software^84^. Before plotting, Shannon’s index was converted in corresponding effective numbers according to^87^. To test for significant differences between the groups, we performed a Kruskal–Wallis test, followed by pairwise Wilcoxon tests using the software R^88^.

## Supplementary Information

Supplementary Figure S1.

Supplementary Tables.

## Data Availability

All 16S rRNA gene sequences produced for this study are available in the Sequence Read Archive (SRA) under accession number PRJNA791286.
