# Supplementary material for: Gut microbiota plasticity in insular lizards under reversed island syndrome
Source: Sci Rep. 2022 Jul 25;12:12682. doi: 10.1038/s41598-022-16955-0 (PMC9314426; doi:10.1038/s41598-022-16955-0)
Supplement: Supplementary file 1 — Supplementary Figure S1. [file 41598_2022_16955_MOESM1_ESM.pdf]

Supplementary information

Gut microbiota plasticity in insular lizards under Reversed Island Syndrome

Maria Buglione[1], Ezio Ricca[1,2], Simona Petrelli[1], Loredana Baccigalupi [2,3], Claudia Troiano[1], Anella Saggese[1], Eleonora Rivieccio[4] and Domenico Fulgione*[1,2]

Affiliations

[1] Department of Biology, University of Naples Federico II, Naples, Italy

[2] Task Force of the Federico II University for microbiome studies, Naples, Italy

[3] Department of Molecular Medicine and Medical Biotechnology, University of Naples Federico II, Naples, Italy

[4] Department of Humanities, University of Naples Federico II, Naples, Italy

Corresponding author*

Domenico Fulgione

Department of Biology, University of Naples Federico II, Via Cinthia 26, 80126 Naples, Italy

E mail address: fulgione@unina.it

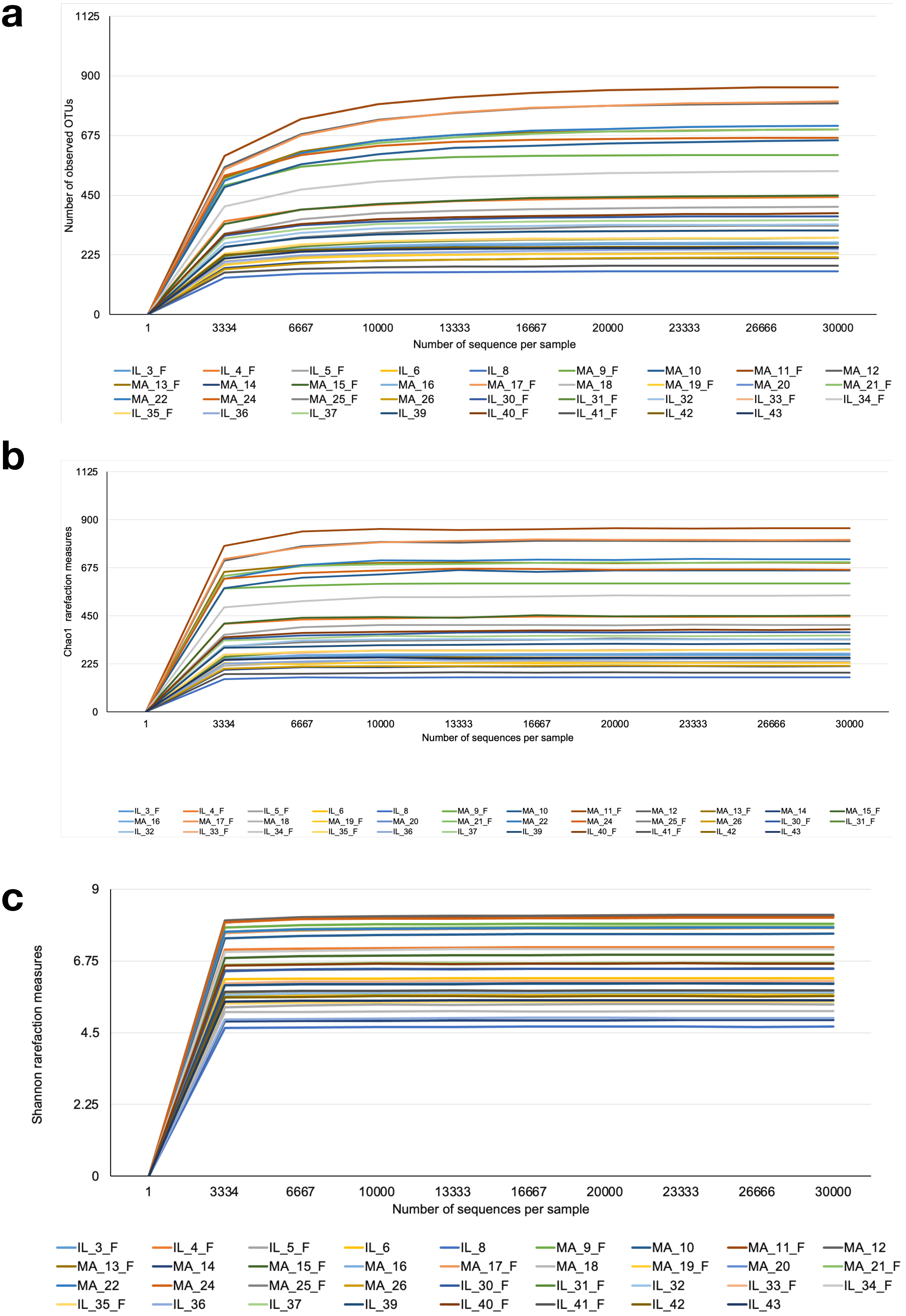
a
Number of observed OTUs
1125
900
675
450
225
0
1 3334 6667 10000 13333 16667 20000 23333 26666 30000
Number of sequence per sample
IL_3_F IL_4_F IL_5_F IL_6 IL_8 MA_9_F MA_10 MA_11_F MA_12
MA_13_F MA_14 MA_15_F MA_16 MA_17_F MA_18 MA_19_F MA_20 MA_21_F
MA_22 MA_24 MA_25_F MA_26 IL_30_F IL_31_F IL_32 IL_33_F IL_34_F
IL_35_F IL_36 IL_37 IL_39 IL_40_F IL_41_F IL_42 IL_43
b
Chao1 rarefaction measures
1125
900
675
450
225
0
1 3334 6667 10000 13333 16667 20000 23333 26666 30000
Number of sequences per sample
IL_3_F IL_4_F IL_5_F IL_6 IL_8 MA_9_F MA_10 MA_11_F MA_12 MA_13_F MA_14 MA_15_F
MA_16 MA_17_F MA_18 MA_19_F MA_20 MA_21_F MA_22 MA_24 MA_25_F MA_26 IL_30_F IL_31_F
IL_32 IL_33_F IL_34_F IL_35_F IL_36 IL_37 IL_39 IL_40_F IL_41_F IL_42 IL_43
c
Shannon rarefaction measures
9
6.75
4.5
2.25
0
1 3334 6667 10000 13333 16667 20000 23333 26666 30000
Number of sequences per sample
IL_3_F IL_4_F IL_5_F IL_6 IL_8 MA_9_F MA_10 MA_11_F MA_12
MA_13_F MA_14 MA_15_F MA_16 MA_17_F MA_18 MA_19_F MA_20 MA_21_F
MA_22 MA_24 MA_25_F MA_26 IL_30_F IL_31_F IL_32 IL_33_F IL_34_F
IL_35_F IL_36 IL_37 IL_39 IL_40_F IL_41_F IL_42 IL_43

Supplementary Fig. S1: a) observed number of OTUs showing number of OTUs *versus* sequence depth for IL (island lizard), IL_F (fed island lizard), MA (mainland lizard) and MA_F (fed mainland lizard). b) **Rarefaction curves for Chao1 and c) Shannon index** showing the microbial community complexes in IL, IL_F, MA and MA_F.
